# Multi-modal and multi-view image dataset for weeds detection in wheat field

**DOI:** 10.3389/fpls.2022.936748

**Published:** 2022-08-22

**Authors:** Ke Xu, Zhijian Jiang, Qihang Liu, Qi Xie, Yan Zhu, Weixing Cao, Jun Ni

**Affiliations:** ^1^College of Agriculture, Nanjing Agricultural University, Nanjing, China; ^2^National Engineering and Technology Center for Information Agriculture, Nanjing, China; ^3^Engineering Research Center of Smart Agriculture, Ministry of Education, Nanjing, China; ^4^Collaborative Innovation Center for Modern Crop Production Co-sponsored by Province and Ministry, Nanjing, China; ^5^College of Artificial Intelligence, Nanjing Agricultural University, Nanjing, China

**Keywords:** multi-modal image, multi-view image, grass weeds detection, wheat field, machine learning, deep learning

## Introduction

Weeds in wheat fields compete with wheat for light, water, fertilizer, and growth space, and therefore are one of the main biohazards that limit the yield and quality formation of wheat (Shiferaw et al., 2013; Singh et al., 2013). Obtaining weed species and location information quickly and accurately is the first step in precise weed control. Existing methods for detecting weeds in wheat fields based on machine learning are highly dependent on the scale and quality of datasets. In particular, deep learning methods usually require massive training samples. However, Multi-modal and Multi-View Image of weed datasets in natural wheat field are very rare currently.

Existing public weed datasets in crop field are shown in Table 1. Compared with other crops such as cotton and sugar beet, the plant space for wheat crop is smaller. Therefore, the background in wheat field is more complex, and the labeling process is more difficult. In addition, the existing weed datasets still have the following drawbacks. First, the number of samples is small, which is difficult to meet the requirements of deep learning. The mode of information is very single and limited. The existing datasets are constructed based on RGB images, which cannot provide a more complete feature space for weed detection. Especially detecting weeds in wheat field, the features extracted from RGB images are easy to detect broadleaf weeds, but it is difficult to detect weeds with similar appearance to wheat. Studies have shown that because of growth competition, height information is an important parameter to discriminate wheat from weeds (Fahad et al., 2015; Xu et al., 2020), and the fusion of RGB image features and height features has become a new method used to improve the efficiency of weed detection. Finally, the existing datasets are mostly based on vertical views for image acquisition (Wu et al., 2021), which is difficult to be applied to weed detection under a complex field background. Leaf overlap and occlusion will have a great impact on detection.

**Table 1:** Public weed datasets in crop field.

**Dataset**	**Purpose**	**Plants**	**Description**	**References**
Dataset of annotated food crops and weed images	Weeds detection and control	Common beet, carrot, zucchini, pumpkin, radish, radish and 8 weed species	1,118 images with 7,853 XML manually annotated annotations	Sudars et al., 2020
A crop/weed field image dataset	Instance segmentation for weeds and plants	Carrot and common weeds in North Germany	60 images with annotations	Haug and Ostermann, 2015
2016 sugar beets dataset	Classification of weeds and plants	Sugar Beet and common weeds in Germany	4-channel multi-spectral images	Chebrolu et al., 2017
Early-crop-weed	Classification of weeds and plants	tomato, cotton, velvetleaf and black nightshade	766 field images of crops in early stage	Espejo-Garcia et al., 2020
Deep weeds	Classification of multiple weeds species	Eight nationally significant weed species	17,509 images with annotations	Olsen et al., 2019
Plant seedlings dataset	Classification of weeds and crops	Maize, wheat, sugar beet and nine weed species	5,539 images with annotations	Giselsson et al., 2017
CNU weeds dataset	Classification of multiple weeds species	21 weeds species in the Republic of Korea	208,477 images with annotations	Vo Hoang et al., 2020
Carrot-weeds	Weeds detection	Carrots and unspecified weeds	39 images with annotations	Lameski et al., 2017
Lincoln Beet	Weeds detection	Sugar beet and unspecified weeds	4,402 images with annotations	Salazar-Gomez et al., 2021
Cobbity Wheat	Weeds detection	Wheat and two weed species	101 images with annotations	Coleman, 2021
Radish Wheat Dataset	Weeds detection	Four growth stages wheat and four weed species	552 images with annotations	Rayner, 2022
Crop and weed	Instance segmentation for weeds and plants	Maize, the common bean and a variety of weeds	2,489 images with annotations	Champ et al., 2020

Therefore, we proposed Multi-modal and Multi-view Image Dataset for Weeds Detection in Wheat Field (MMIDDWF) that can be used for deep learning. The dataset contains wheat, broad-leaf weed, and grass weed images of two modes and nine views, and aims to provide a public weed dataset to promote the development of weed detection methods in wheat field.

## Value of the data

- A multimodal image dataset will be provided for weed detection in open wheat field, including an RGB image and a depth image of the same scene. Compared with a single RGB image, a depth image can provide three-dimensional structure features for weed detection in wheat field, which is helpful to solve the problem of detecting grass weeds.
- The dataset also contains multi-view images. Images from nine views can provide a more complete feature space for weed detection in open wheat field, thus helping to solve weed detection problems under a complex background such as leaf occlusion and overlapping.

## Materials and methods

### Experiment design and image acquisition

Experiments on wheat and weeds were carried out from December 2017 to April 2021 at the demonstration base of the National Engineering and Technology Center for Information Agriculture in Rugao county, Nantong City, Jiangsu province, China. Weeds were not controlled during field management, and seeds of six weed species commonly associated with wheat were randomly sown to simulate weed growth in the open field. *Alopecurus aequalis, Poa annua, Bromus japonicus*, and *E*. *crusgalli* are grass weeds; *Amaranthus retroflexus* and *C*. *bursa-pastoris* are broad-leaf weeds; species composition was similar to that of actual weed species in wheat fields.

**Figure 1:**
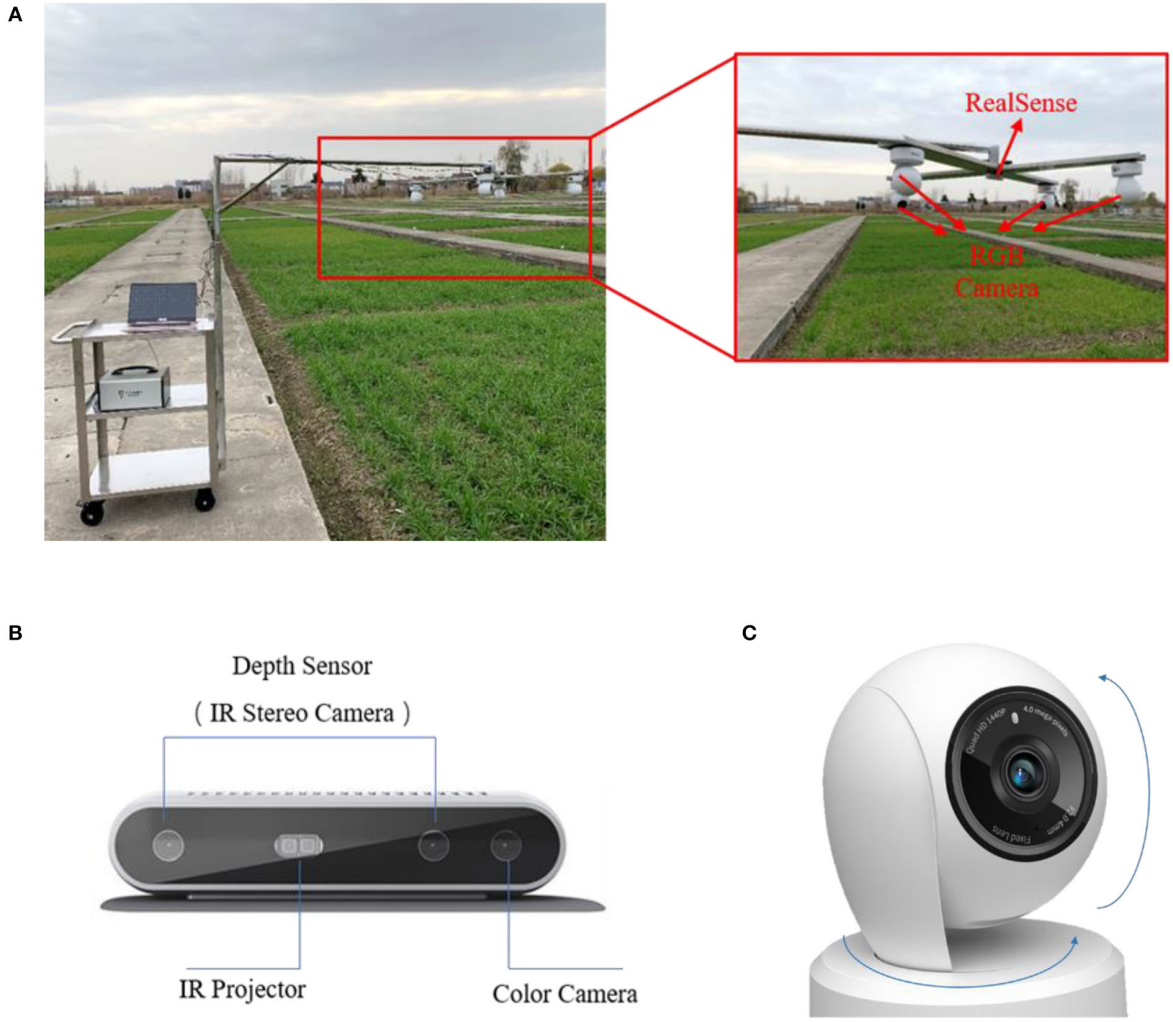
**(A)** Image acquisition equipment, **(B)** Intel^®^ RealSense™ Depth Camera D415, and **(C)** TL-IPC44AN-4camera.

The data were collected at the peak of weed occurrence in wheat fields, i.e., in the tillering and jointing stages. The acquisition equipment is shown in Figure 1. The RGB and depth images were acquired using Intel^®^ RealSense™ Depth Camera D415 (Integrated Electronics Corporation, Santa Clara, CA, United States), an RGB-D camera that adopts active infrared stereo vision technology. Infrared stereo cameras generate depth images, and the color sensor generates RGB images, both with a resolution of 1,280 × 720.

RGB and depth field images under natural conditions were obtained during the wheat tillering and jointing stages. Multi-view images were collected with TL-IPC44AN-4(TP-Link Corporation, Shenzhen, China) cameras in four positions at angles of 15 and 30° horizontally. Image collection was conducted from 9 a.m. to 4 p.m. under clear and windless weather conditions. The camera was 70 cm above the crop canopy. Images were transmitted to a computer in real time *via* USB 3.0.

### Image annotation and dataset production

**Figure 2:**
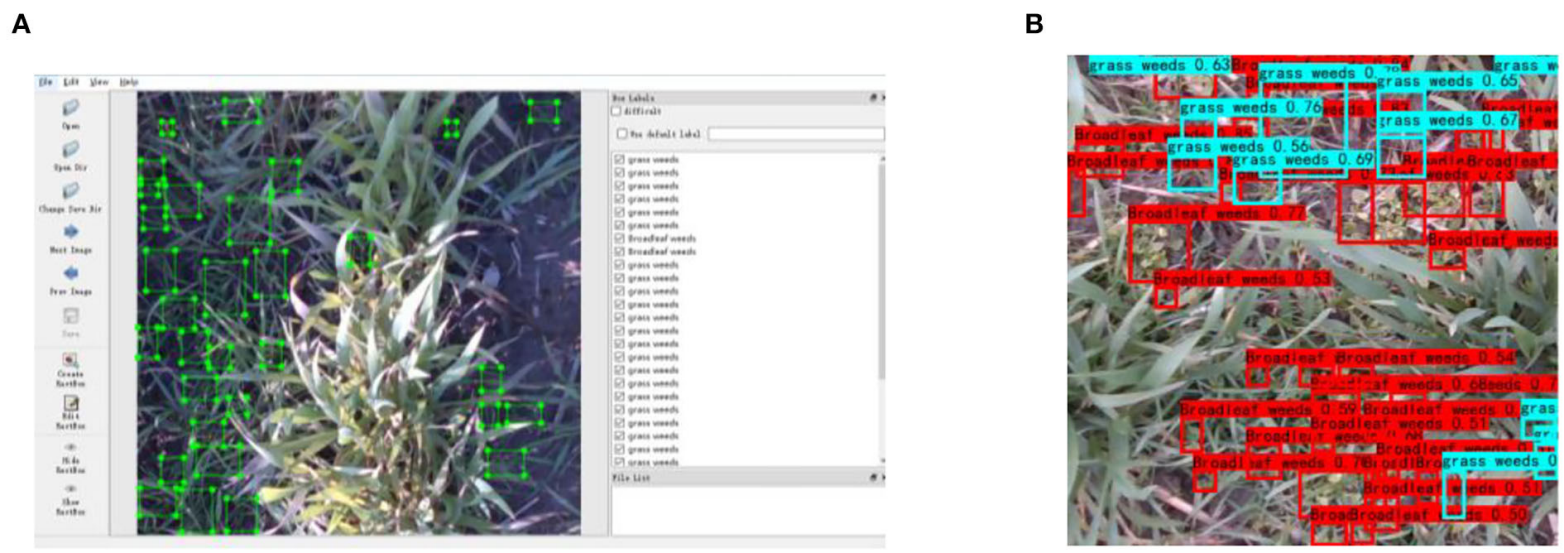
**(A)** Labeling of grass and broadleaf weeds in wheat fields using LabelImg and **(B)** weed detection result in wheat field.

The original depth information is less representative. In particular, feature extraction from depth images with a convolutional neural network generates feature maps of distance rather than geometric structures with physical significance. Therefore, single-channel depth images were transformed to three-channel images by re-encoding the original images to make them more representative and structurally similar to RGB images (Gupta et al., 2014). The three channels of re-encoded images are phase, height above ground, and angle with gravity, and re-encoded images are referred to as PHA images. For the image re-encoding method, refer to Xu et al. (2021). Therefore, the multimodal image dataset in MMIDDWF includes three parts: RGB images, single-channel depth images corresponding to RGB images, and PHA images obtained by recoding depth images. In MMIDDWF, each type includes 1,288 images measuring 500 × 500 pixels. The multi-view image dataset contains 692 images, including 79 RGB images from vertical view, 79 vertical depth images corresponding to vertical view RGB images, and 534 images from eight other views. The details of the dataset are shown in Table 2. LabelImg is employed to annotate broadleaf and grass weeds in images as shown in Figure 2A, and the annotation information of RGB images corresponds to that of depth and PHA images. Figure 2B shows the detection results of weeds in wheat fields based on the multimodal dataset (Xu et al., 2021), which achieved precise detection results in an open wheat field by proposing a dual-channel convolutional neural network and fusing multimodal information.

**Table 2:** Details about MMIDDWF.

**Wheat varieties information**	**Planting row spacing**	**Weeds species information**	
**Experiment design**			
Shengxuan No.6, Sumai No.8, Yangmai No.16 and Yangmai No.23	20, 35,and 50 cm	Four grass weeds, two broadleaf weeds and other native weeds in wheat fields	
**Multi-modal image dataset**			
Camera	Angle	Type and number of images	Image size
Intel^®^ RealSense™ Depth Camera D415	vertical horizontal plane 90°	1,288 RGB images and 1,288 PHA images	500 × 500
**Multi-view image dataset**			
Camera	Angle	Type and number of images	Image size
TL-IPC44AN-4 camera	with the horizontal plane is 15° and 30°	534 RGB images	2,560 × 1,440
Intel® RealSense™ Depth Camera D415	vertical horizontal plane 90°	79 RGB images and 79 depth images	720 × 1280

## Data availability statement

The datasets presented in this study can be found in online repositories. The names of the repository/repositories and accession number(s) can be found below: https://github.com/cocococoxu/MMDWWF.

## Author contributions

JN, YZ, KX, and WC designed the research. KX, ZJ, QL, and QX conducted the experiment. KX and ZJ analyzed the data and wrote the manuscript. All authors have read and approved the final version of the manuscript.

## Funding

This study was supported by the National Natural Science Foundation of China (Grant No: 31871524), Modern Agricultural Machinery Equipment and Technology Demonstration and Promotion of Jiangsu Province (Grant No: NJ2021-58), Primary Research and Development Plan of Jiangsu Province of China (Grant No: BE2021304), and Six Talent Peaks Project in Jiangsu Province (Grant No: XYDXX-049).

## Conflict of interest

The authors declare that the research was conducted in the absence of any commercial or financial relationships that could be construed as a potential conflict of interest.

## Publisher's note

All claims expressed in this article are solely those of the authors and do not necessarily represent those of their affiliated organizations, or those of the publisher, the editors and the reviewers. Any product that may be evaluated in this article, or claim that may be made by its manufacturer, is not guaranteed or endorsed by the publisher.
